# At the Hospital Gates

**Published:** 1888-01-28

**Authors:** 


### AT THE HOSPITAL GATES.

DEAR, our journey's end is this—
Here we part,
Clasp me, give me kiss for kiss,
Heart to heart.
I'm a coward, nigh despair,
Death perchance awaits me there,
Spite of all that these can dare—
Art and care.

Nay, good-bye, I will be brave—
You must smile—
No! I will not dread the grave
Yet awhile.
Trust me, I am fearless, bold,
As in careless days of old;
It boots not that the hand you hold,
Still is cold.

Of sweet memory and thought,
I've a store;
Treasured up to feast on. What
Need I more?
Sacred memories, ne'er outworn,
Of our joyous wedding morn,
And of griefs together borne—
Flower and thorn—

These shall my companions be,
Faithful friends!
They shall strengthen, comfort me,
Till this ends—
Till our fears are provéd vain,
This day's loss to-morrow's gain,
And I come to you again,
Free from pain.

Or if—if—(I do not fear,
No, not I;
Yet, just let me say it, dear)
If I die,
Though our sky should overcast,
Life's bright grains run out too fast,
I will think, though life is past,
Love will last.

